# Clinical features of Q fever vertebral osteomyelitis: case reports and literature review

**DOI:** 10.3389/fmed.2026.1938416

**Published:** 2026-09-15

**Authors:** Tao Zhang, Hong Liang Gao, Hua Liu, Song Kai Li

**Affiliations:** Department of Spine Surgery, The 940th Hospital of Joint Logistics Support Force of Chinese PLA, Lanzhou, Gansu, China

**Keywords:** *Coxiella burnetii*, Q fever, spinal infection, spondylodiscitis, vertebral osteomyelitis

## Abstract

**Background:**

This study aims to characterize Q fever vertebral osteomyelitis based on our experience and literature review.

**Methods:**

By reviewing all reported cases of Q fever vertebral osteomyelitis since the 21st century, we characterized the exposure history, clinical presentations, treatment strategies, and prognostic outcomes.

**Results:**

Fifty cases of Q fever vertebral osteomyelitis were identified. The mean age was 63.4 ± 15.7 years, and 86% (43/50) of the participants were male. Of the 50 patients, 21 had a history of contact with cattle, sheep, or wild animals, and of the 43 patients, 26 had vascular comorbidities. Low back pain (39/45) was the most common symptom. The lumbar spine was the most susceptible region, with the L4–L5 segment being the most frequently involved site. Of the 47 patients, 39 had multivertebral infections, and the majority also had psoas abscesses (25/41). The positive rate of next-generation sequencing (NGS; 7/7) in spinal lesion tissues was the highest, followed by that of polymerase chain reaction (PCR) detection (26/30). The most common treatment regimen was doxycycline combined with hydroxychloroquine (31/49). The proportions of patients with fever (36% vs. 5.9%) and elevated C-reactive protein (CRP; 90% vs. 41.7%) levels were higher in the group of patients with vertebral osteomyelitis and those with concurrent vascular defects (*p* < 0.05). However, separate comparative analyses of the two subgroups showed no significant difference in overall outcomes (*p* > 0.05).

**Conclusion:**

Q fever vertebral osteomyelitis should be included in the differential diagnosis, particularly in older men and patients with concurrent vascular defects. PCR or NGS performed on local specimens provides critical advantages for rapid pathogen identification. Doxycycline combined with hydroxychloroquine remains the most used treatment regimen at present.

## 1 Introduction

In recent years, the incidence of spinal infections has increased due to the growing number of older patients with chronic diseases, as well as those with immunocompromised conditions, those using steroids, drug abusers, and patients undergoing invasive spinal procedures (1, 2). The specific pathogens causing spinal infections include not only *Mycobacterium tuberculosis* and *Brucella* species but also common causative bacteria of pyogenic vertebral osteomyelitis, such as *Staphylococcus aureus* (57–58%), *Escherichia coli* (1.05–16%), *Streptococcus* spp. (6.7–11%), and anaerobic bacteria (3%) (3, 4). However, cases of vertebral osteomyelitis caused by *Coxiella burnetii* are extremely rare.

*Coxiella burnetii*, a Gram-negative obligate intracellular bacterium, is the pathogen of Q fever. Typically, Q fever presents in two clinical forms: the acute disease, which is more common and often asymptomatic, and persistent focalized infection (chronic Q fever), which occurs in 4–5% of patients and is generally associated with a poor prognosis (5). The manifestations of chronic Q fever include endocarditis, vasculitis, and osteoarthritis (5, 6). Among the osteoarticular manifestations, spondylodiscitis or vertebral osteomyelitis is the most common form (7).

*Coxiella burnetii* infection is common in humans and various animal species; however, it has not been fully recognized in many countries, thereby posing a significant threat to human and animal health (8). Due to its rapid spread and high infectivity, *C. burnetii* infection is a public health, veterinary, and economic concern. At present, Q fever vertebral osteomyelitis may be an under-recognized clinical condition (9), and research on Q fever vertebral osteomyelitis is primarily based on individual case reports, with no case series studies. Therefore, we analyzed the clinical features of Q fever vertebral osteomyelitis to enhance clinicians’ comprehensive understanding of this disease.

## 2 Methods

### 2.1 Case 1

A 66-year-old man, with no travel history, presented with a 6-month history of low back pain in the absence of constitutional symptoms such as fever, night sweats, weight loss, or nocturnal back pain. The comorbidities included hypertension, a history of left lower extremity deep vein thrombosis, and inferior vena cava filter implantation. The patient lived in an urban area and had no contact with cattle or sheep. The body temperature was 36.3 °C, and there was tenderness upon palpation of the lumbar spine. The visual analog scale (VAS) score for the intensity of lumbar pain, graded on a 0–10 scale, was 5 out of 10, indicating moderate pain. Laboratory investigations revealed a white blood cell count (WBC) of 2.81×10^9^/L, C-reactive protein (CRP) of 22 mg/L, erythrocyte sedimentation rate (ESR) of 37 mm/h, and procalcitonin (PCT) of 0.24 ng/mL. The serological test for Brucella and the interferon-gamma release assay (IGRA) were both negative. Magnetic resonance imaging (MRI) confirmed the presence of chronic osteomyelitis at the L4–L5 level, accompanied by psoas abscesses. Computed tomography (CT) scans revealed bone destruction of the L4 and L5 vertebrae (Figure 1). No abnormalities were found on echocardiography and vascular ultrasound examinations. The preoperative diagnosis was lumbar osteomyelitis, although the specific pathogen remained unidentified. Due to severe low back pain that significantly affected his daily life activities and work, the patient underwent debridement and oblique lumbar interbody fusion (OLIF) surgery. Histopathological examination revealed inflammatory infiltration accompanied by proliferative granulation tissue. Quantitative real-time polymerase chain reaction (qPCR) detected *C. burnetii* DNA in the spinal lesion tissues; however, the sample culture results were negative. Although serological testing can provide valuable information on the stage of Q fever, this testing method was not available at our hospital during the diagnostic process. Eventually, the patient was diagnosed with Q fever vertebral osteomyelitis, and a chemotherapy regimen of doxycycline (200 mg/day) combined with ciprofloxacin (1,000 mg/day) was planned for at least 18 months. A follow-up Lumbar MRI examination after 6 months of chemotherapy confirmed significant improvement in the previous observed vertebral erosion, with no evidence of acute inflammation or masses. At the last follow-up, 6 months after the surgery, the patient’s clinical condition in terms of infection remained stable.

**Figure 1:**
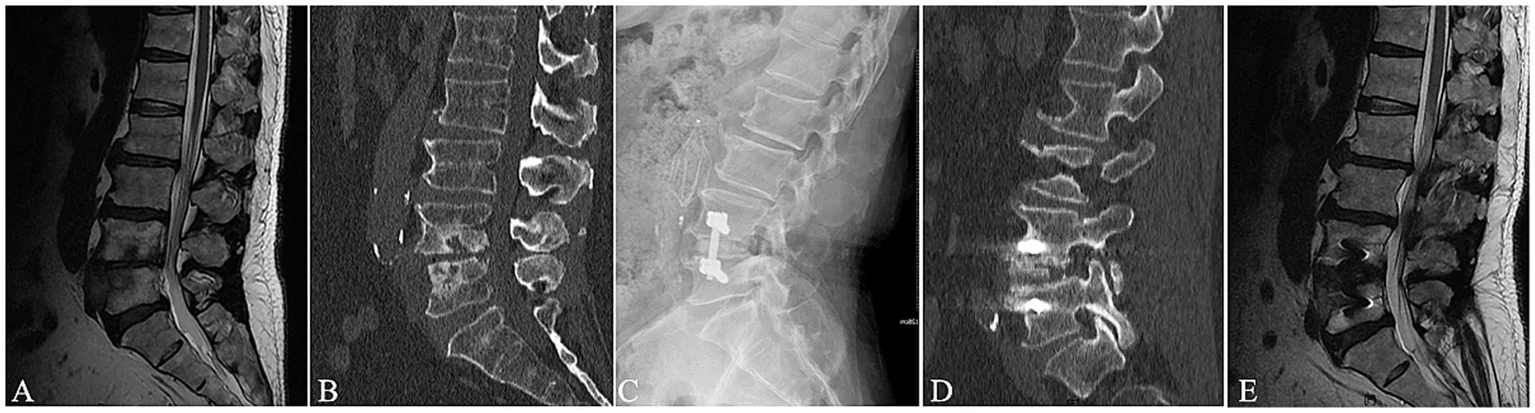
**(A)** Lumbar MRI confirmed chronic osteomyelitis at the L4–L5 level. **(B)** CT scans revealed bone destruction at the L4 and L5 vertebrae. **(C)** X-ray imaging at 6 months postoperatively demonstrated proper positioning of the internal fixation device and bone graft. **(D)** CT scan at 6 months postoperatively revealed successful fusion of the intervertebral bone graft. **(E)** MRI at 6 months postoperatively showed normal isointensity of the L4 and L5 vertebrae.

### 2.2 Case 2

A 56-year-old man with no travel history presented with persistent low back pain for 12 months, accompanied by pain in the left lower limb and intermittent claudication, but without fever, night sweats, or weight loss. His comorbidities included a history of lumbar spinal stenosis. The patient was a cattle attendant (in contact with cattle and sheep) and lived in a rural area. The body temperature was 36.3 °C. Physical examination revealed lumbar tenderness, hypoesthesia of the left lower leg, and a positive Lasègue sign. The VAS scores for the intensity of pain in the lumbar region and the lower limbs were 6 out of 10 and 5 out of 10, respectively. Laboratory investigations revealed a WBC count of 18.2×10^9^/L, CRP of 1.75 mg/L, ESR of 3 mm/h, and PCT of 0.4 ng/mL. The serological tests for Brucella and IGRA were both negative. MRI confirmed the presence of chronic osteomyelitis at the L4–L5 level, along with epidural and right psoas abscesses. CT scans revealed bone destruction at the endplates of the L4 and L5 vertebrae (Figure 2). Echocardiography ruled out endocarditis. The preoperative diagnosis was lumbar osteomyelitis, and because the patient presented with obvious low back and leg pain, debridement and transforaminal lumbar interbody fusion (TLIF) surgery was performed. Histopathological examination revealed necrotic foci in the L4–L5 intervertebral disc sample. To diagnose this unexplained infection, targeted next-generation sequencing (tNGS) testing and culture were performed on samples collected from the spinal lesion tissue. The tNGS test results revealed that *C. burnetii* and *Pseudomonas aeruginosa* were detected in specimens collected from the spinal lesion; however, the culture results were negative. Eventually, the patient received a chemotherapy regimen of doxycycline (200 mg/day) combined with ciprofloxacin (1,000 mg/day) for a planned duration of at least 18 months. After 9 months of postoperative chemotherapy, the patient’s clinical symptoms had completely resolved, and he was still undergoing further treatment and follow-up.

**Figure 2:**
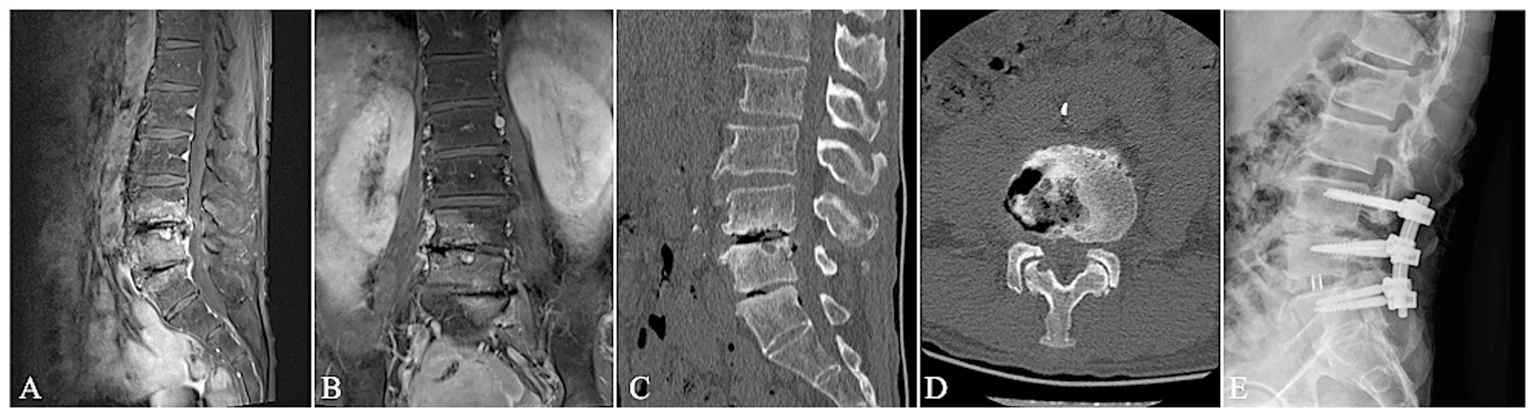
**(A,B)** Lumbar MRI revealed chronic osteomyelitis at the L4–L5 level, along with an epidural abscess and right psoas abscess. **(C,D)** CT scans showed bone destruction at the endplates of the L4 and L5 vertebrae. **(E)** The postoperative X-ray imaging demonstrated proper positioning of the internal fixation device and bone graft.

### 2.3 Case 3

A 57-year-old man with a history of percutaneous endoscopic lumbar discectomy presented with low back pain, night sweats, and nocturnal pain for the past 7 months, accompanied by pain and numbness in both lower extremities, but without fever or weight loss. Histopathological examination after lumbar discectomy at another hospital revealed chronic granulomatous inflammation; therefore, the patient received 5 months of empirical anti-tuberculosis treatment postoperatively. However, the low back pain and pain in the bilateral lower extremities did not show significant improvement. The patient was a sheep attendant who lived in a rural area. The body temperature was 36 °C. There was tenderness upon palpation of the lumbar spine, hypoesthesia of the bilateral lower legs, and a positive Lasègue sign. The VAS score for the intensity of lumbar pain indicated a moderate level of 6 out of 10. Laboratory tests revealed a WBC count of 5.37×10^9^/L, CRP of 3.6 mg/L, ESR of 4 mm/h, and PCT of 0.02 ng/mL. The serological tests for Brucella and IGRA were both negative. No pathogenic microorganisms were detected in the blood culture. MRI revealed vertebral osteomyelitis and an epidural abscess at the L5-S1 segment. CT scans showed bone destruction at the posterior margin of the L5 vertebra and the superior margin of the S1 vertebra (Figure 3). No abnormalities were found on echocardiography or vascular echography. Due to ineffective anti-tuberculosis therapy and worsening low back pain, the patient underwent debridement and TLIF surgery. Histopathological examination showed chronic suppurative inflammation with necrosis, accompanied by proliferating granulomas. qPCR detected *C. burnetii* DNA in the spinal lesion tissues, but the culture results were negative. The diagnosis of Q fever vertebral osteomyelitis was made, and treatment with doxycycline (200 mg/day) combined with ciprofloxacin (1,000 mg/day) was initiated for a period of 18 months. During the 6-month follow-up after chemotherapy and surgery, the patient’s clinical symptoms completely resolved, and the MRI demonstrated that the epidural abscess had completely disappeared. After the 24-month follow-up, the patient had fully recovered clinically and radiologically after discontinuation of the treatment.

**Figure 3:**
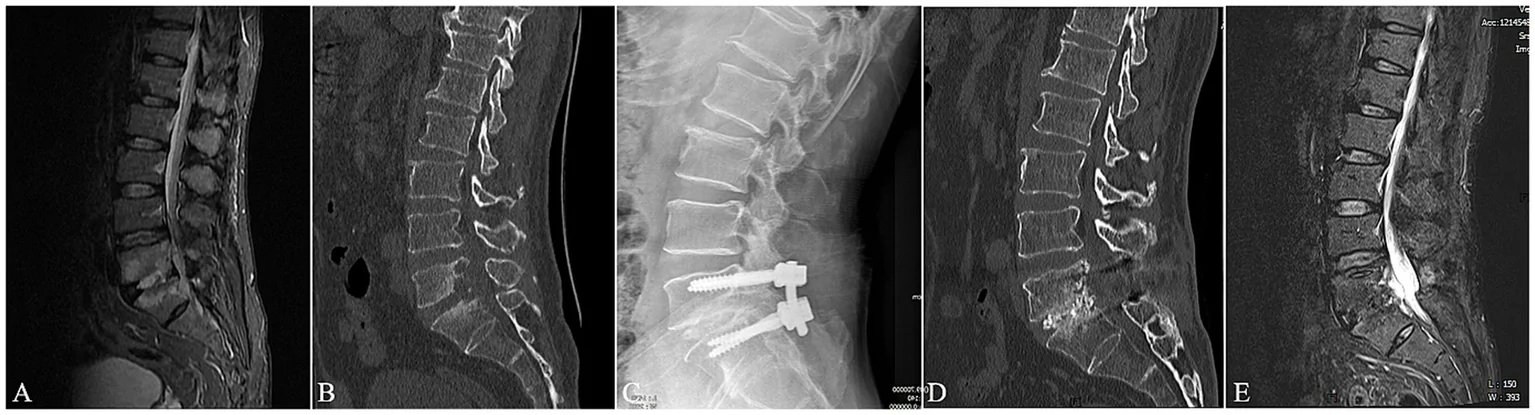
**(A)** Lumbar MRI confirmed chronic osteomyelitis at the L5–S1 level with an expanding epidural abscess. **(B)** Lumbar CT scan showed bone destruction at the posterior margin of the L5 vertebra and the superior margin of the S1 vertebra. **(C)** X-ray imaging at 24 months postoperatively demonstrated proper positioning of the internal fixation device and bone graft. **(D)** CT scan at 24 months postoperatively revealed successful fusion of the intervertebral bone graft. **(E)** MRI at 24 months postoperatively showed complete disappearance of hyperintensity within the L5 and S1 vertebrae.

### 2.4 Study design

The study followed the Preferred Reporting Items for Systematic Reviews and Meta-Analyses (PRISMA) guidelines (10) to identify studies and describe the screening, inclusion, and exclusion processes in this review.

#### 2.4.1 Literature search

A systematic literature search was performed in PubMed, MEDLINE, Web of Science, Scopus, and Wanfang databases, using keywords such as “*Coxiella burnetii*,” “Q fever,” “Spinal infection,” “Vertebral osteomyelitis,” and “Spondylodiscitis.” The researchers independently conducted searches using the Boolean operators “AND” and “OR” and screened the literature published between 1 January 2000 and 30 April 2026. Additional relevant studies were identified through manual screening of references.

#### 2.4.2 Inclusion and exclusion criteria

The diagnostic criteria for Q fever vertebral osteomyelitis (11, 12) were based on the presence of any one of the following items: (1) a positive culture result from spinal lesion samples; (2) a positive PCR (or NGS) test result from spinal lesion samples; and (3) clinical and imaging evidence of spinal infection accompanied by positive serology with phase *I* IgG antibodies titers of ≥1:800.

This review included studies reporting demographic features, exposure history, symptoms, complications, diagnostic methods, treatment strategies, and outcomes of patients with Q fever vertebral osteomyelitis. Only articles written in English or Chinese with available abstracts were included. Expert opinions, clinical trials, animal studies, comments, letters, meta-analyses, practice guidelines, duplicate publications, and abstracts from scientific meetings were excluded.

#### 2.4.3 Data extraction and quality evaluation

The two researchers independently screened the literature in strict accordance with the inclusion and exclusion criteria. Any discrepancies were resolved through consensus with the third author. The extracted information included the age and gender of the patients, exposure history, comorbidities, clinical presentations, the location of vertebral infection, related diagnostic tests, treatment strategies, and clinical outcomes.

### 2.5 Statistical analysis

Statistical calculations were performed using SPSS version 20.0 (IBM Corp., Armonk, NY, USA). Continuous variables were analyzed using independent samples t-tests. Categorical variables were analyzed using Fisher’s exact test. A two-sided *p*-value of <0.05 was considered statistically significant.

## 3 Results

A total of 33 articles (13–45) were included, involving clinical case data from 50 patients (Figure 4). The demographic, epidemiological, medical history, presenting symptoms, laboratory tests, imaging, histopathology, treatment, and follow-up outcomes are detailed in Tables 1, 2.

**Figure 4:**
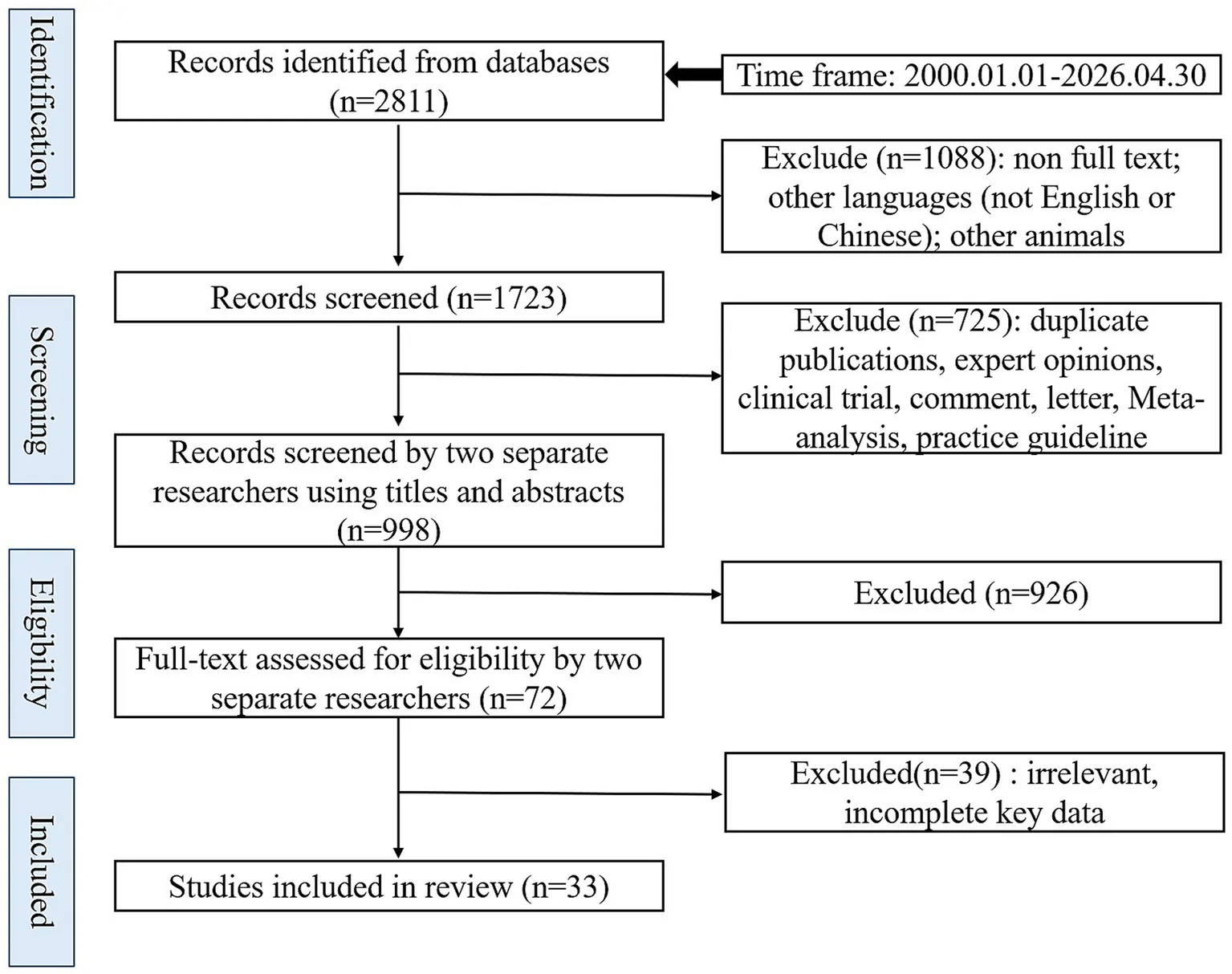
The flowchart for article screening.

**Table 1:** Demographic, epidemiological and clinical characteristics of cases.

Case no.	Reference	Year	Age/ gender	Residence	Risk factors	Co-morbidity	Presenting symptoms	Duration of symptoms	Vascular defect/ Endocarditis status
1	Current case		66/M	City	No	Hypertension, history of inferior vena cava filter implantation	Low back pain	6-month	Lower extremity deep venous thrombosis (filter implantation)
2	Current case		56/M	Countryside	Cattle attendant, contact with cattle and sheep	Lumbar spinal stenosis	Low back pain, left lower limb pain and intermittent claudication	12-month	No
3	Current case (13)	2025	57/M	Countryside	Sheep attendant, contact with sheep	History of percutaneous endoscopic lumbar discectomy	Low back pain, night sweats, nocturnal pain, lower extremities pain and numbness	7-month	No
4	C. Nourse (14)	2004	4/M	Countryside (Dairy farm)	Cow contact	History of infection and surgical intervention in the right foot and chest wall	Back pain		No
5	C. Nourse (14)	2004	7/M		Spent time on farm with goats	No		8-month	
6	C. Nourse (14)	2004	61/F			Uterine cancer			
7	C. Nourse (14)	2004	39/M	Countryside	Dairy farmer				
8	C. Nourse (14)	2004	76/F	Countryside					
9	P. S. G. d. Costa (15)	2006	67/M		No		Fever		
10	G. Breton (16)	2007	66/M		Contact with farm animals	History of coronary artery bypass	Lumbar pain and fever (38.5°)	2-month	Infrarenal AAA (38 mm)
11	G. Breton (16)	2007	65/M			Coronary artery disease	Low back pain, weight loss		AAA (55 mm) (Resection and bypass reconstruction)
12	C. Landais (17)	2007	64/M			Rheumatoid arthritis, chronic hepatitis C, osteoporotic vertebral fracture, myocardial infarction, and arteriopathy with stent	Lumbar pain, night fever (38°)	1-month	infrarenal AAA (20 mm)
13	C. Landais (17)	2007	47/M			No	Right S1 sciatic pain with left leg paresthesia	2-month	No
14	M. E. O’Donnell (18)	2007	66/M	Countryside		Left hemisphere cerebrovascular accidents, history of elective aortobifemoral bypass surgery	Severe back pain and fever (37.8°)		Bilateral aortoiliac aneurysms (Resection and bypass reconstruction)
15	V. Merhej (19)	2012	70/M		Consumption of artisanal Corsican goat cheese	History of endovascular aortic repair	Abdominal and lumbar pain, anorexia and weight loss	2- month	Infrarenal AAA (51 mm) (Resection and bypass reconstruction)
16	P. D. M. Aerts (20)	2013	77/M				Intermittent claudication		Infected AAA (60 mm); stenoses in both iliac arteries and occlusion of the superficial femoral arteries
17	E. Heldenberg (21)	2015	54/M	Prison inmate	No	Tobacco abuse	Back pain and intermittent claudication	2-year	Infrarenal AAA (45 mm); the right external iliac artery and the left common, internal and external iliac arteries were occluded (Stent graft insertion)
18	A. Galy (22)	2016	92/M	City	No	Hypertension, diabetes, coronary artery disease	Lumbar pain	3-year	Infrarenal AAA (78 mm) (Aortobiiliac venous allograft)
19	M. Gaudé (23)	2016	55/M	Countryside	No direct contact with animals except his dog	Hypertension, obesity	Chronic low back pain	Several months	No
20	J. Jayet (24)	2016	91/M			Hypertension, diabetes mellitus, obesity	Fever and chronic low back pain		Infrarenal AAA (78 mm) (Resection and bypass reconstruction)
21	P. A. Leahey (25)	2016	57/M		No contact with animals but possible consumption of unpasteurized milk	Emphysema, tobacco use	Lower back pain with intermittent fevers		Infrarenal AAA (81 mm) (Endovascular aneurysm repair)
22	M. Michel (26)	2016	64/M			Graft repair of a ruptured aortic aneurysm	Low back pain	9-month	AAA (Graft repair)
23	W. Stokes (27)	2016	67/M		No	Previous history of AAA open repair, chronic renal disease secondary to hypertension, tobacco use	Lower back and abdominal pain	4-year	Perirenal AAA (118*143 mm) (redo repair of AAA)
24	A. Virk (28)	2017	74/M	Countryside (Farmer)	Exposures included deer hunting, raising livestock, and drinking unpasteurized milk	Hypertension, coronary artery disease, history of AAA graft repair and abdominal wall hernia repair	Back pain		Infected AAA (Resection and bypass reconstruction)
25	A. Virk (28)	2017	60/F	Countryside	Lived on a farm with animal exposure 20 years ago	History of AAA graft repair, hypertension, hyperlipidemia, chronic obstructive pulmonary disease, coronary artery disease, and pulmonary carcinoid status post–wedge resection	Back pain, weight loss, and gait instability		Infected AAA (Resection and reconstruction)
26	A. Virk (28)	2017	57/F	Countryside	Contact with cattle and sheep	Metastatic colon adenocarcinoma			AAA (43*40 mm) (Graft repair and reconstruction)
27	A. Virk (28)	2017	67/M	Countryside	Surrounding livestock, drinking unpasteurized goat milk, hunting wild game	History of AAA graft repair	Low back pain, sweats, and malaise	6-month	Infected AAA (40*21*55 mm) (Graft explantation and reconstruction)
28	E. Meriglier (29)	2018	86/M		No		Weakness, back pain		No
29	E. Meriglier (29)	2018	66/M				Back pain, pain in one leg		No
30	E. Meriglier (29)	2018	59/M		No		Back pain		No
31	N. Mongardon (30)	2018	66/M	Countryside	No	History of AAA graft repair	Lumbar pain and low-grade fever	2-month	AAA (Graft explantation and reconstruction)
32	P. Lundy (31)	2019	74/M			Coronary artery disease, lumbar stenosis, history of AAA graft repair	Low back pain and radiation down in right lower extremity	1-month	AAA
33	S. Browning (32)	2020	55/M	Countryside	Meat packing and abattoir work, work on a cattle farm, hunting foxes, kangaroos and rabbits	Chronic hepatitis C, child Pugh B liver cirrhosis, history of cervical surgery	Thoracic back pain, weight loss	6-month	No
34	S. Browning (32)	2020	58/M	Countryside	Cattle farmer	Acute Q fever	Lumbosacral pain	4-month	No
35	V. Waelbers (33)	2020	69/F		Contact with sheep	History of AAA graft repair	Lower back pain and fever		Infected AAA (Graft explantation and reconstruction)
36	K. Dorfman (34)	2021	78/F			Dyslipidemia, osteoarthritis, osteoporosis, vertebroplasty(T12)	Epigastric pain, anorexia, and weight loss	2-month	No
37	N. Ghanem-Zoubi (35)	2021	80/M		No	Hyperlipidaemia, hypertension, chronic obstructive pulmonary disease and history of AAA graft repair, tobacco use	Low back pain, fever and weight loss	2-month	Infected AAA
38	N. Ghanem-Zoubi (35)	2021	72/M			Hypertension, diabetes mellitus, coronary artery disease and paroxysmal atrial fibrillation, tobacco use	Low back pain and haematuria		Infrarenal AAA (24 mm) (Graft repair)
39	N. Ghanem-Zoubi (35)	2021	66/M			Hypertension, chronic kidney disease, AAA, and history of coronary artery bypass grafting, tobacco use	Weakness and weight loss	2-month	Infected AAA (Debridement and reconstruction)
40	N. Ghanem-Zoubi (35)	2021	78/F			Vertebroplasty (T12)	Right upper abdominal pain, weight loss and fever	2-month	
41	A. S. L. Lim (36)	2021	74/M			Hypertension, diabetes mellitus, and hyperlipidaemia	Low back pain	3-month	Infrarenal AAA (73*76*57 mm) (Graft repair)
42	L. Vincente (37)	2022	72/M	Countryside	Contact with goats	Hypertension	Back pain, loss of strength in lower limbs	12-month	No
43	S. Yang (38)	2023	66/M	Countryside (Farmer)		No	Low back pain, numbness and lower limb weakness	36-month	No
44	JH. SHEN (39)	2024	58/M		No	History of lumbar intervertebral disc surgery, hypertension and osteoporosis	Low back pain, fever, radicular pain and numbness of lower extremity	2-month	No
45	O. Mangkalamanee (40)	2024	64/M	Countryside (Farmer)	Goat dairy farmer, contact with goats	No	Lower back pain, tightness in right hip and inner thigh	12-month	No
46	J. Chen (41)	2025	66/M	Countryside (Farmer)	Contact with sheep	No	Low back pain, stiffness of lower limbs	5-month	No
47	Y. Liu (42)	2025	57/M		Contact with sheep manure	Diabetes, hypertension and atrophic gastritis	Lower back pain, irregular fever (38.5°), fatigue	12-month	AAA (aortic repair)
48	F. Masoch (43)	2025	69/M			HIV infection, history of cross-femoral bypass for lower limb arteriopathy	Low back pain		Lower limb arteriopathy
49	M. Song (44)	2025	52/M		Pigeon breeder, avian exposure		Low back pain, burning sensation in the left lower limb, numbness on the right thigh	12-month	
50	M. Xin (45)	2025	60/M	Countryside (Farmer)		Hypertension, hyperlipidemia	Low back pain, lower limbs weakness, and radiating pain in the left hip	2-month	No

M: Male; F: Female; AAA: Abdominal aortic aneurysm.

**Table 2:** Imaging, laboratory, treatment and follow-up outcomes of cases.

Case No.	Reference	Involved Vertebra	Complications	Inflammatory markers	Relevant diagnostic tests		Histopathology	Treatment	Time of follow up/ Outcomes
					Serological test	Microbiological tests			
1	Current case	L4-L5 (Debridement+ OLIF)	Psoas abscess	WBC2.81 × 10^9^/L; CRP22mg/L; ESR37 mm/h; PCT0.24 ng/ml	Not available	spinal lesion sample cultures (−), qPCR (+)	Inflammatory infiltration and proliferation granuloma	Dox + Cip (18 months, recommendation)	6 months/ Favorable outcome
2	Current case	L4-L5 (Debridement+ TLIF)	Epidural and psoas abscess	WBC18.2 × 10^9^/L; CRP1.75 mg/L; ESR3 mm/h; PCT0.4 ng/ml	Not available	spinal lesion sample cultures (−), tNGS (*Coxiella burnetii*, *Pseudomonas aeruginosa*, +)	Necrotic foci	Dox + Cip (18 months, recommendation)	9 months/ Favorable outcome
3	Current case (13)	L5-S1 (Debridement+ TLIF)	Epidural abscess	WBC5.37 × 10^9^/L; CRP3.6 mg/L; ESR4 mm/h; PCT0.02 ng/ml	Not available	Blood cultures (−); spinal lesion sample cultures (−), qPCR (+)	Chronic suppurative inflammation with necrosis, proliferation granuloma	Dox + Cip (18 months, recommendation)	24 months/ Recovery
4	C. Nourse (14)	T10	Epidural abscess and left foot abscess	WBC, CRP, ESR: normal	IgG (*I*) 1:2560; IgG (*II*) 1:2560	Vertebral biopsy cultures (−); surgical specimens PCR (+)	Granulomatous inflammatory process with tight aggregates of histiocytes and open granulomas containing central necrosis and dystrophic calcification	Cla + Cot (6 months); Dox + Rif (1 year); Cip (4 months); Dox + Hyd	5 years/ Recovery
5	C. Nourse (14)	L3	Right hip chronic recurrent osteomyelitis		IgG (*I*) 1:1600	Cell cultures of L3 (+)		Oxa (3 months); Dox (2 years) + Rif (6 months)	6.5 years/ Recovery
6	C. Nourse (14)	Lumbar spondylitis			IgG (*I*) 1:800				
7	C. Nourse (14)	L5			CFT (*I*) 1:128; CFT (*II*) 1:512		No organisms detected	Lin + Tet (11 weeks)	7 years/ Recovery
8	C. Nourse (14)	T12-L1	Psoas abscess		CFT (*I*) 1:512; CFT (*II*) 1:1024		Caseous material, histiocytes, lymphocytes, and multinucleate giant cells	Rif + Iso + Eth (2 years)	Continued presence of symptoms
9	P. S. G. d. Costa (15)			WBC9.0 × 10^9^/L; CRP50mg/L	IgG (*I*) 1:1024; IgG (*II*) 1:1024			Dox + Cip + Hyd	Recovery
10	G. Breton (16)	L3-L4	Psoas abscess	ESR44 mm/h; CRP41 mg/L	IgG (*I*) 1:1600; IgG (*II*) 1:12800	Blood cultures (−); Abscess aspiration cultures (−), PCR (+)	Chronic inflammation and altered polynuclear leukocytes	Dox + Hyd (3 years)	3 years/ Favorable outcome
11	G. Breton (16)	L4-L5	Psoas abscess	ESR88 mm/h; CRP71 mg/L	IgG (*I*) 1:800; IgG (*II*)1:1600	Blood cultures (−); Abscess aspiration cultures (−), PCR (+)	Polynuclear leukocytes	Dox + Ofl + Hyd	2 months/ Worsening
12	C. Landais (17)	L2-3, L4-L5	Paravertebral, psoas and epidural abscess	WBC: normal; ESR77 mm/h; CRP33 mg/L	IgG (*I*) 1:400; IgG (*II*)1:800	Blood cultures (−); disco-vertebral biopsy cultures (−), PCR (+); paravertebral fluid using cell culture (+)	Purulent paravertebral fluid	Dox + Rif (2 weeks); Dox + Hyd	2 months/ Favorable outcome
13	C. Landais (17)	L5-S1	Epidural abscess	WBC, ESR, CRP: normal	IgG (*I*) 1:1600; IgG (*II*) 1:3200	Blood cultures (−); abscess puncture cultures (−); abscess and serum PCR (+)	Inflammatory lesions with tuberculoid granuloma	Dox + Hyd	1 year/ Favorable outcome
14	M. E. O’Donnell (18)	L3-L4, L1, L2 (Debridement)	Psoas abscess; femoral lymphadenopathy; prosthetic graft sepsis and peri graft abscess	WBC, ESR: normal; CRP20 mg/L	IgG (*I*) 1:1280; IgG (*II*) 1:1280	Paravertebral hematoma cultures (−); thrombus PCR (+)		Dox + Cip (for life)	21 months/ Recovery
15	V. Merhej (19)	L2-3	Psoas abscess	WBC7.0 × 10^9^/L; CRP34mg/L	IgG (*I*) 1:12800; IgG (*II*) 1: 25600	Puncture liquid cultures (−), PCR (+); psoas abscess using cell culture (+)	Neutrophils and mononuclear cells, predominantly macrophages	Dox + Hyd	6 months/ Favorable outcome
16	P. D. M. Aerts (20)	Destruction of several lumbar vertebrae		WBC5.3 × 10^9^/L; CRP12mg/L; ESR22 mm/h	IgG (*I*) 1:640	Blood PCR (+)		Dox + Hyd	Died after AAA reconstruction surgery
17	E. Heldenberg (21)	L3-L4 (Debridement)	Psoas abscess		1:3200	Blood cultures (−); abscess aspiration cultures (*Gemella morbillorum*+)		Van, Dox + Hyd + Met+ Rif (for life)	
18	A. Galy (22)	L3 (Debridement)	Psoas abscess	WBC: normal; CRP10mg/L	IgG (*I*) 1:8192; IgG (*II*) 1:4096	Abscess cultures (−)	Non-caseous necrosis and granulomas, without giant cells	Dox + Hyd (18 months)	6 months, lost to follow-up / Favorable outcome
19	M. Gaudé (23)	L4-L5	No	CRP2.9 mg/L	IgG (*I*) 1:2048; IgG (*II*) 1:4096	Blood and disco-vertebral cultures (−), PCR (−)		Dox + Hyd (10 months)	10 months/ Recovery
20	J. Jayet (24)	L3 (Debridement)	Paravertebral and psoas abscess	WBC: normal; CRP16mg/L	IgG (*I*) 1:8000		No sign of granulomatosis	Dox + Hyd (18 months)	20 months/ Recovery
21	P. A. Leahey (25)	L3-L4 (Debridement and fusion)	Psoas and epidural abscess		IgG (I) 1:2048; IgG (*II*) 1:1024	Blood cultures (−); hematoma tissue cultures (−), PCR (−); Vertebral tissue cultures (−), RT-PCR (+)	Granuloma, and intracellular Gram-negative coccobacilli	Dox + Hyd (for life)	3 months/ Favorable outcome
22	M. Michel (26)	L3-L4	Psoas abscess			Blood cultures (−); L3 biopsy specimen cultures (−), PCR (−); psoas abscess PCR (+)	Epithelioid granulomatous inflammatory reaction surrounding areas of necrosis	Dox + Hyd	
23	W. Stokes (27)	L2-L3, L1 (Debridement)	Epidural abscess	CRP156.8 mg/L	IgG (*I*) 1:2048; IgG (*II*) 1:1024	Blood cultures (−); vascular tissue cultures (−), PCR (+);	No signs of infection or granulomas	Dox + Hyd	22 months/ Favorable treatment outcome but died from pulmonary embolism
24	A. Virk (28)	L2-L3, L1 (Debridement)	Psoas abscess, epidural abscess (T10-L3)	WBC6.4 × 10^9^/L; CRP36mg/L; ESR53 mm/h	IgG (*I*) 1:1024; IgG (*II*) 1:256	Abscess cultures (−); blood cultures (−); Vertebra tissue cultures (−), PCR (+), cell culture (+)	Granulomas with neutrophilic inflammation	Dox + Cip, Dox + Hyd	55 days/ Died from acute myocardial infarction
25	A. Virk (28)	L2-L4 (Debridement and fixation)	Psoas phlegmon		IgG (*I*) 1:1024; IgG (*II*) 1:256	Vertebral tissue cultures (−), PCR (+)	Chronic inflammation, giant cell reaction, calcification, and focal necrosis	Dox + Cip (for life)	6.5 years/ Favorable outcome
26	A. Virk (28)	L2-L4 (Debridement)			IgG (*I*) 1:2048; IgG (*II*) 1:4096	Vertebra tissue and aorta cultures (+), PCR (+)	Dense fibrosis with chronic inflammation	Dox + Cip, Dox + Hyd (1 year), Dox (59 months)	71 months/ Favorable treatment outcome but died from metastatic colon cancer
27	A. Virk (28)	L2-L3 (Debridement)	Prevertebral and psoas abscess	WBC9.5 × 10^9^/L; CRP279mg/L; ESR20 mm/h	IgG (*I*) 1:1634; IgG (*II*) 1:8192	Abscess cultures (−)	Inflammation with negative stains for bacteria, fungi, and mycobacteria	Van+ Cef + Met (6 weeks), Dox + Hyd (24 months)	
28	E. Meriglier (29)	L2-L3	Psoas abscess		IgG (*I*) < 1:800	Abscess cultures (+), PCR (+)		Dox + Hyd (18 months)	>19 months/ Favorable outcome
29	E. Meriglier (29)	L4-L5, L5-S1	Paravertebral abscess		IgG (*I*) > 1:800	Vertebra tissue PCR (+)		Dox + Hyd (18 months)	>19 months/ Favorable outcome
30	E. Meriglier (29)	L5-S1	Paravertebral abscess, epidural localization		IgG (*I*) > 1:800	Vertebra tissue PCR (+)		Dox + Hyd (18 months)	>19 months/ Favorable outcome
31	N. Mongardon (30)	L2-L3 (Debridement)	Paravertebral and psoas abscesses	WBC, CRP, ESR: normal	IgG (*I*) 1:2048; IgG (*II*) 1:256	Abscess cultures (−)		Dox + Hyd	6 months/ Favorable outcome
32	P. Lundy (31)	L5-S1 (Debridement)	Prevertebral abscess		IgG (*I*) 1:32000	Disc tissue cultures (−); pelvic tissue PCR (+)		Van (6 weeks), Dox + Hyd (12–18 months)	Favorable outcome
33	S. Browning (32)	T8-T9, T10-T11	Epidural abscess	CRP16mg/L	IgG (*I*) 1:800; IgG (*II*) 1:6400	Blood cultures (−); vertebra tissue cultures (−), qPCR (+)	No evidence of active inflammation or neoplasm	Dox + Hyd, Tri/Sul	20 months/ Relapse
34	S. Browning (32)	L5-S1		CRP13mg/L	IgG (*I*) 1:12800; IgG (*II*) 1:102400	Blood cultures (−); vertebra tissue cultures (−), disc tissue qPCR (+)	Fragmented bone and marrow with trilinear haematopoiesis	Dox + Hyd (18 months)	12 months/ Favorable outcome
35	V. Waelbers (33)	L4-L5, L3 (Debridement)	Presacral and epidural abscess	WBC4.2 × 10^9^/L; CRP 129.9 mg/L	IgG (*I*) 1:4096; IgG (*II*) 1:16384	Blood cultures (−); vertebra tissue cultures (−), pus cultures (−), PCR (+)		Dox + Hyd (18 months)	5 months/ Favorable outcome
36	K. Dorfman (34)	T11-T12	Paravertebral abscess	WBC13.5 × 10^9^/L; CRP72.4 mg/L	IgG (*I*) 1:3200; IgG (*II*) 1:1600	Blood cultures (−); pus aspiration cultures (−), PCR (+)		Dox + Hyd (18 months)	9 months/ favorable outcome
37	N. Ghanem-Zoubi (35)	L4	Psoas abscess	CRP110mg/L	IgG (*I*) 1:6400	Blood cultures (*Streptococcus pneumoniae*+); vertebra tissue cultures (*Klebsiella pneumoniae*, *Prevotella intermedia* and *Fusobacterium* sp.+), PCR (−); blood RT-PCR (−)	Polymorphonuclear inflammation	Lev (3 months), Dox + Hyd (24 months) + Mox	42 months/ Recovery
38	N. Ghanem-Zoubi (35)	L3		WBC, CRP: normal	IgG (*I*) 1:1600	Blood cultures (−), PCR (−)		Dox + Hyd (18 months)	18 months/ Favorable outcome
39	N. Ghanem-Zoubi (35)	L5	Psoas abscess	CRP18.5 mg/L, WBC: normal	IgG (*I*) 1:12800; IgG (*II*) 1:200	Blood cultures (−), PCR (−); pus aspiration cultures (−), PCR (−); aorta tissue RT-PCR (+)	Fragments of fibrin with reactive lymph nodes	Dox + Hyd, Dox + Lev (18–24 months)	6 months/ Favorable outcome
40	N. Ghanem-Zoubi (35)	T11-T12	Right-sided pleural effusion, paravertebral abscess	CRP230mg/L	IgG (*I*) 1:25600	Pleural fluid culture (−); pus aspiration PCR (+); blood PCR (−)		Dox + Hyd (18 months)	12 months/ Favorable outcome
41	A. S. L. Lim (36)	L4-L5 (Debridement and fixation)	Psoas and paravertebral abscess	CRP276mg/L; ESR32 mm/h	IgG (*I*) 1:3200; IgG (*II*) 1:3200	Blood cultures (−); abscess aspiration PCR (−), cultures (−); disc cultures (*MRSA*+)	Granulomatous inflammation	Ert, Amo/ Cla + Lev (6 months); Iso + Rif + Eth + Lin; Dox + Hyd (for life)	24 months/ Favorable outcome
42	L. Vincente (37)	L2-L3 (Debridement and fixation)	Paravertebral, psoas and epidural abscess		IgG (*I*) 1:2048; IgG (*II*) 1:2048	Biopsy specimen cultures (−); blood cultures (−); pus cultures (−)		Mer (8 weeks); Dox + Hyd (18 months)	12 months
43	S. Yang (38)	L1-L2 (Debridement and fixation)	Epidural abscess	CRP1.05 mg/L; ESR9.9 mm/h		Blood cultures (−); vertebra biopsy tissue mNGS (+)	Inflammatory granuloma	Rif + Dox + Lev (18 months)	16 months/ Recovery
44	JH. SHEN (39)	L4-L5 (Debridement and fixation)	Psoas abscess	WBC5.89 × 10^9^/L; CRP78 mg/L; ESR 80 mm/h		Disco-vertebral tissue cultures (−), mNGS (+)		Van+ Cip (6 days), Dox + Hyd	1 month/ Favorable outcome
45	O. Mangkalamanee (40)	L3-L4, L4-L5, L5-S1 (Debridement and fixation)	Epidural and psoas abscess	WBC: normal	Brucella IgM and IgG (+); *C. burnetii* IgM and IgG (−)	Bone tissue cultures (−), PCR (+); blood cultures (Brucella spp.+), PCR (*B. melitensis*+);	No granuloma or tumor cells	Gen + Rif + Dox (1 week), Rif + Dox + Hyd	2 months/ Favorable outcome
46	J. Chen (41)	L3-L4 (Debridement and fixation)		WBC7.3 × 10^9^/L; CRP2.6 mg/L; ESR 20 mm/h; PCT0.02 ng/mL		Vertebra biopsy cultures (−), nanopore gene detection (*C. burnetii*, *Candida albicans* +); disc sample cultures (−), mNGS (+)	Granulation tissue hyperplasia, cellulose necrosis and inflammatory cell infiltration	Flu+ Lev+ Rif; Lev+ Dox + Rif (22 days), Dox + Rif	Favorable outcome
47	Y. Liu (42)	L2-L3 (Abscess drainage)	Psoas abscess	WBC5.05 × 10^9^/L; CRP 66.31 mg/L; ESR 77 mm/h	Interferon-gamma release assay (+)	Blood cultures (−); L3 vertebra biopsy cultures (−), mNGS (+)		Dox + Hyd	8 months/ Favorable outcome
48	F. Masoch (43)	L3-L4		CRP 42 mg/L	IgG (*I*) 1:4093	Blood cultures (−); disco-vertebral biopsy cultures (−), PCR (−)		Dox + Hyd	12 months/ Favorable outcome
49	M. Song (44)	L3-L5	Epidural abscess	WBC5.77 × 10^9^/L; CRP11.77 mg/L; ESR 16 mm/h		Blood cultures (−); vertebra aspirate tissue mNGS (+)	Chronic inflammatory infiltrates and necrotic foci at L5	Van+ Dox (1 month); Dox (17 months)	6 months/ Favorable outcome (with spinal nerve impairment)
50	M. Xin (45)	L4-L5 (Debridement)	No	WBC8.02 × 10^9^/L; CRP46.97 mg/L; ESR 88 mm/h; PCT0.044 ng/mL	IgG (*I*), IgG (*II*): (+)	Lesion tissue mNGS (+)	Acute and chronic inflammatory cell infiltration	Mox (2 weeks) + Min + Hyd	Favorable outcome

WBC: White blood cell; CRP: C-reactive protein; ESR: Erythrocyte sedimentation rate; PCR: Polymerase chain reaction; qPCR: Quantitative real-time polymerase chain reaction; RT-PCR: Real-time polymerase chain reaction; tNGS: Targeted next-generation sequencing; mNGS: Metagenomic next-generation sequencing; OLIF: Oblique lumber interbody fusion; TLIF: Transforaminal lumber interbody fusion; MRSA: methicillin-resistant *Staphylococcus aureus*; CFT: Complement fixation titer; Oxa: Oxacillin; Dox: Doxycycline; Lin: Lincomycin; Tet: Tetracycline; Rif: Rifampicin; Iso: Isoniazid; Eth: Ethambutol; Min: minocycline; Hyd: Hydroxychloroquine; Mox: Moxifloxacin; Cla: Clarithromycin; Cot: Cotrimoxazole; Cip: Ciprofloxacin; Flu: Fluconazole; Lev: Levofloxacin; Ofl: Ofloxacine; Met: Metronidazole; Van: Vancomycin; Cef: Cefepime; Tri/Sul: trimethoprim/ sulfamethoxazole; Ert: Ertapenem; Amo/ Cla: Amoxicillin/clavulanic acid; Lin: Linezolid; Mer: Meropenem; Gen: Gentamicin.

The mean age was 63.4 ± 15.7 years (range 4–92), and 86% (43/50) of the patients were male. Among these patients, 38% (19/50) clearly lived in the countryside, and 42% (21/50) had a definite history of contact with cattle, sheep, or wild animals. Additionally, 60.5% (26/43) of the patients also had concurrent vascular comorbidities, such as abdominal aortic aneurysm (AAA) or arteriopathy (Table 3).

**Table 3:** Epidemiology, clinical symptoms, laboratory tests, treatment regimen and outcomes characteristics of Q fever vertebral osteomyelitis.

Characteristics	Cases (50)	Median/ Percentage
Age, mean ± SD, years (Range)	63.4 ± 15.7	66 (4 ~ 92)
Male, n/N (%, [95% CI])	43/50	86.0%, [76.0% ~ 96.0%]
Residence		
Countryside, n/N (%, [95% CI])	19/50	38.0%, [24.1 ~ 51.9%]
City, n/N (%, [95% CI])	2/50	4.0%, [−1.6% ~ 9.6%]
Not reported, n/N (%, [95% CI])	28/50	56.0%, [41.7% ~ 70.3%]
Exposure risk factors		
Yes, n/N (%, [95% CI])	21/50	42.0%, [27.8% ~ 56.2%]
No, n/N (%, [95% CI])	10/50	20.0%, [8.5% ~ 31.5%]
Not reported, n/N (%, [95% CI])	19/50	38.0%, [24.1% ~ 51.9%]
Symptoms		
Back pain, n/N (%, [95% CI])	39/45	86.7%, [76.3% ~ 97.0%]
Fever, n/N (%, [95% CI])	11/45	24.4%, [11.4% ~ 37.5%]
Weight loss, n/N (%, [95% CI])	8/45	17.8%, [6.2% ~ 29.4%]
Vascular defect (%, [95% CI])	26/43	60.5%, [45.2% ~ 75.7%]
AAA, n/N (%, [95% CI])	24/26	92.3%, [81.3% ~ 103.3%]
Arteriopathy, n/N (%, [95% CI])	1/26	3.8%, [−4.1% ~ 11.8%]
Phlebocholosis (filter implantation), n/N (%, [95% CI])	1/26	3.8%, [−4.1% ~ 11.8%]
Vertebra		
Isolated vertebra infection, n/N (%, [95% CI])	8/47	17.0%, [5.9% ~ 28.2%]
Multiple vertebrae infections, n/N (%, [95% CI])	39/47	83.0%, [71.8% ~ 94.1%]
Complications		
Psoas abscess, n/N (%, [95% CI])	25/41	61.0%, [45.4% ~ 76.6%]
Epidural abscess, n/N (%, [95% CI])	15/41	36.6%, [21.2% ~ 52.0%]
Inflammatory markers		
WBC > 9.5 × 109/L, n/N (%, [95% CI])	2/25	8.0%, [−3.4% ~ 19.4%]
CRP > 5 mg/L, n/N (%, [95% CI])	26/35	74.3%, [59.1% ~ 89.5%]
ESR > 15 mm/h, n/N (%, [95% CI])	13/20	65.0%, [42.1% ~ 87.9%]
Serological test, IgG (*I*) ≥ 1:800, n/N (%, [95% CI])	34/39	87.2%, [76.2% ~ 98.2%]
Microbiological tests		
Blood cultures (*C. burnetii +*), n/N (%, [95% CI])	0/24	0.0, [0.0 ~ 0.0]
Tissue cultures (*C. burnetii +*), n/N (%, [95% CI])	2/37	5.4%, [−2.2% ~ 13.0%]
PCR (*C. burnetii +*) in tissues, n/N (%, [95% CI])	26/30	86.7%, [73.8% ~ 99.6%]
NGS (*C. burnetii +*) in tissues, n/N (%, [95% CI])	7/7	100%, [100% ~ 100%]
Antibiotic regimen		
Dox + Hyd, n/N (%, [95% CI])	31/49	63.3%, [49.3% ~ 77.3%]
Dox + Cip, n/N (%, [95% CI])	5/49	10.2%, [1.4% ~ 19.0%]
Dox + Rif, n/N (%, [95% CI])	2/49	4.1%, [−1.7% ~ 9.8%]
Others, n/N (%, [95% CI])	11/49	22.4%, [10.3% ~ 34.6%]
Follow up time, mean ± SD, months (Range)	20.5 ± 22.6	12 (1 ~ 84)
Outcomes		
Favorable outcome, n/N (%, [95% CI])	30/45	66.7%, [52.3% ~ 81.0%]
Recovery, n/N (%, [95% CI])	10/45	22.2%, [9.6% ~ 34.9%]
Died, n/N (%, [95% CI])	2/45	4.4%, [−1.8% ~ 10.7%]
Ineffective treatment or Relapse, n/N (%, [95% CI])	3/45	6.7%, [−0.9% ~ 14.2%]

95% CI: 95% confidence interval; AAA: Abdominal aortic aneurysm; WBC: White blood cell; CRP: C-reactive protein; ESR: Erythrocyte sedimentation rate; PCR: Polymerase chain reaction; NGS: Next-generation sequencing; Dox+ Hyd: Doxycycline+ Hydroxychloroquine; Dox+ Cip: Doxycycline+ Ciprofloxacin; Dox+ Rif: Doxycycline+ Rifampicin.

The most common clinical symptom of Q fever vertebral osteomyelitis was moderate or uncontrollable back pain (86.7%, 39/45), followed by fever. Eighty-three percent (39/47) of the patients had multivertebral infections, and the majority (61%, 25/41) also had psoas abscesses. Among the affected vertebrae, the L3 vertebra was the most common, but among the involved spinal levels, the L4–L5 level was the most common (Figures 5, 6). In the inflammatory marker test, elevated CRP and ESR were frequently observed, but abnormal WBC counts were relatively rare. The positive rates of blood culture and tissue culture for *C. burnetii* were extremely low. Thus, the diagnosis of Q fever vertebral osteomyelitis was primarily based on PCR/NGS detection in the spinal lesion tissues or positive serology for phase I IgG antibodies; among these diagnostic methods, NGS detection had the highest positive rate (100%, 7/7). In terms of treatment strategies, the most common regimen was doxycycline combined with hydroxychloroquine (63.3%, 31/49), followed by doxycycline combined with ciprofloxacin (10.2%, 5/49). The mean follow-up time was 20.5 ± 22.6 (range 1–84) months, and a majority of the patients achieved favorable outcomes or recovery (Table 3).

**Figure 5:**
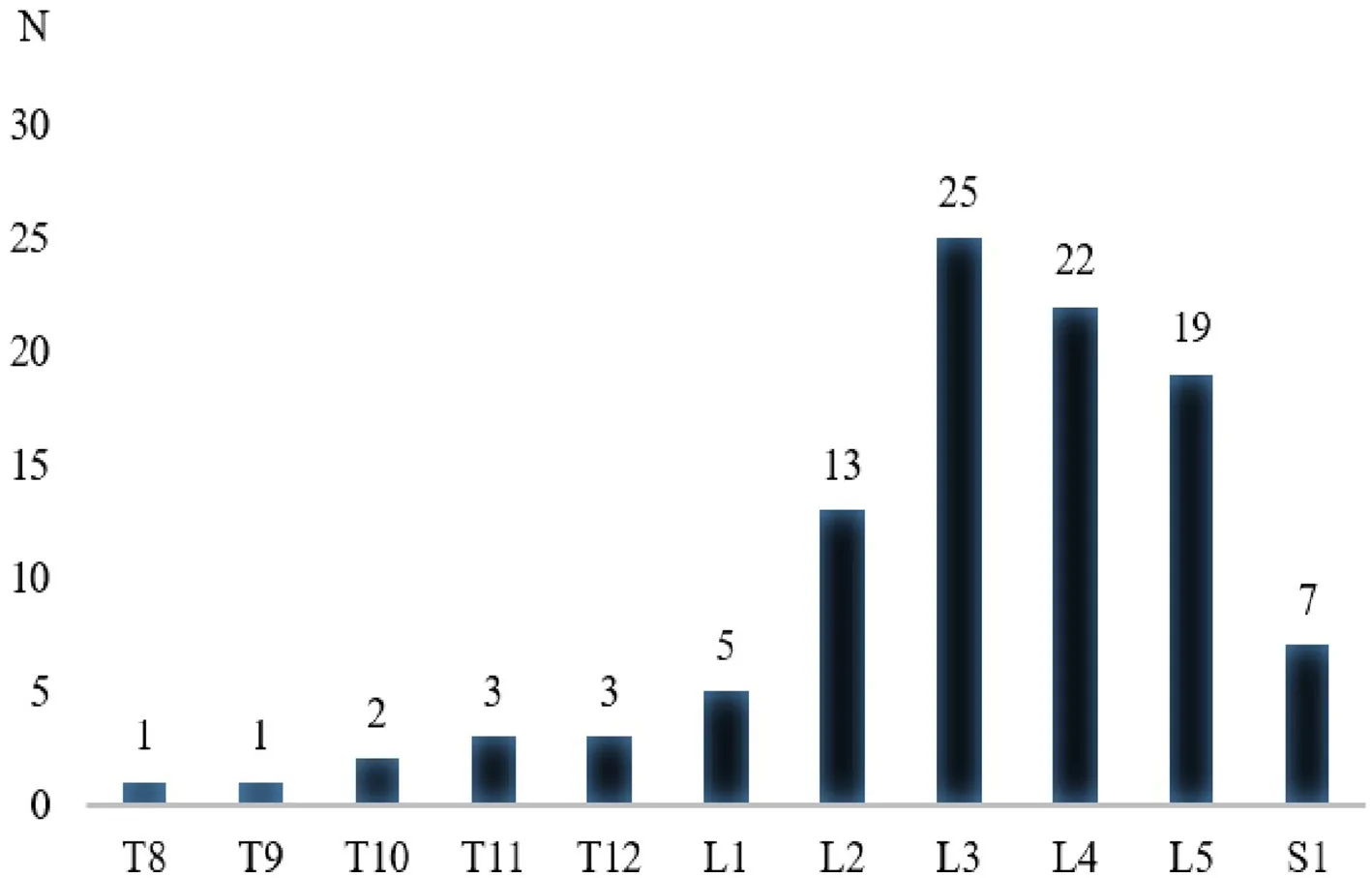
The bar chart illustrates the vertebrae distribution of Q fever vertebral osteomyelitis.

**Figure 6:**
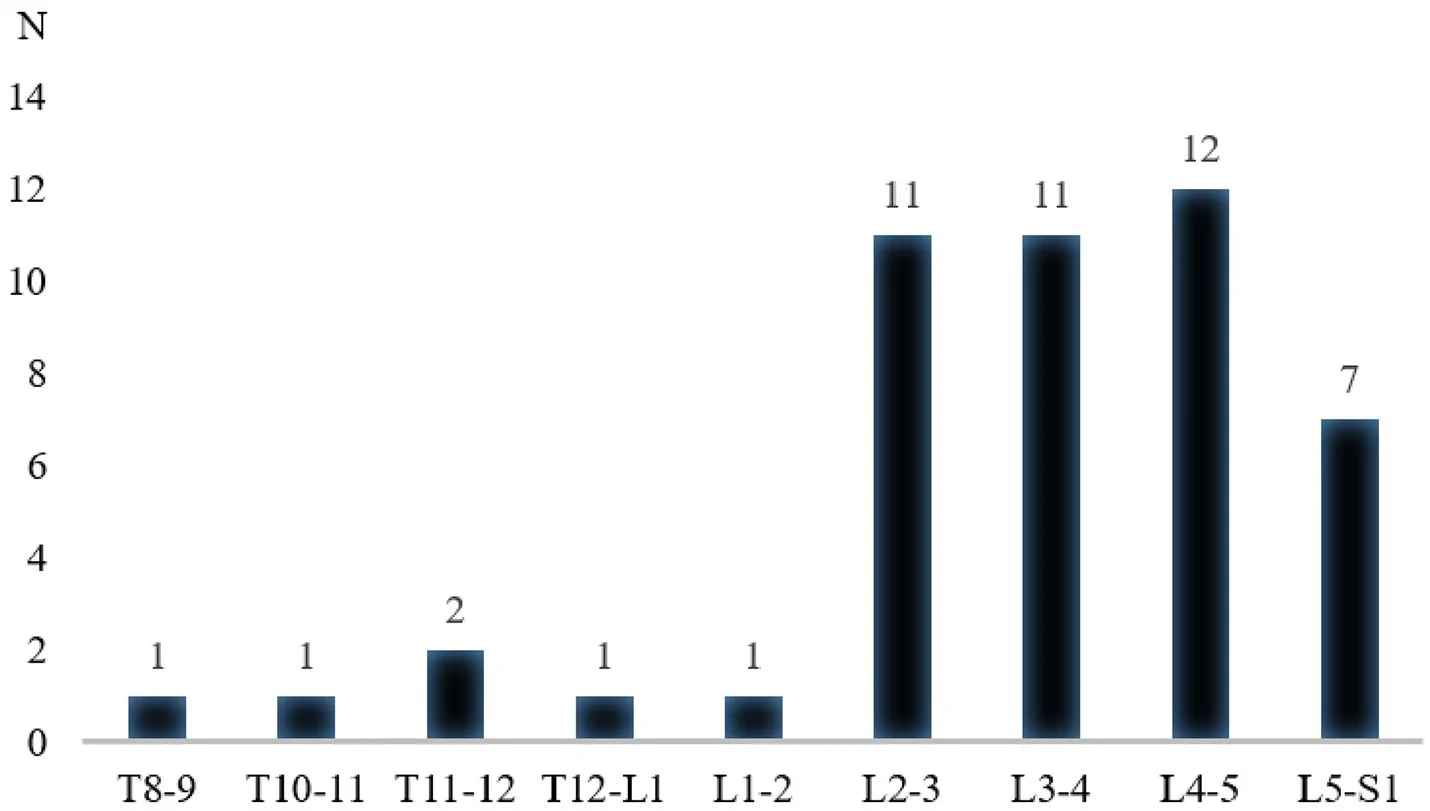
The bar chart illustrates the segment distribution of Q fever vertebral osteomyelitis.

Based on these characteristics, the patients were divided into surgical and non-surgical groups according to whether they underwent debridement surgery. In addition, they were classified into the vertebral osteomyelitis with the vascular defects group and the simple vertebral osteomyelitis group according to whether they had combined vascular lesions. Subsequently, the clinical characteristics of each group of patients were compared. When comparing the clinical features of the vertebral osteomyelitis with vascular defects group and the simple vertebral osteomyelitis group, statistically significant differences were found in age, fever, and CRP level between the two groups (*p* < 0.05). Among them, patients in the simple vertebral osteomyelitis group were younger and had a lower proportion of fever and abnormal CRP levels, while there were no significant differences in other clinical features and outcomes (Table 4).

**Table 4:** Comparing the clinical characteristics, treatment strategies and outcomes of patients with Q fever vertebral osteomyelitis accompanied by vascular defects with those of patients with simple Q fever vertebral osteomyelitis.

Item	Vertebral osteomyelitis with vascular defect (*n* = 26)	Simple vertebral osteomyelitis (*n* = 17)	*P* Value
Age, mean±SD, years	68.6 ± 9.2	59.2 ± 17.0	0.024
Male, n/N (% [95% CI])	23/26 (88.5%, [75.3% ~ 101.6%])	16/17 (94.1%, [81.6% ~ 106.6%])	0.642
Fever, n/N (% [95% CI])	9/25 (36.0%, [15.8% ~ 56.2%])	1/17 (5.9%, [−6.6% ~ 18.4%])	0.031
Elevated CRP (>5 mg/L), n/N (% [95% CI])	18/20 (90.0%, [75.6% ~ 104.4%])	5/12 (41.7%, [8.9% ~ 74.4%])	0.006
Elevated ESR (>15 mm/h), n/N (% [95% CI])	9/11 (81.8%, [54.6% ~ 109.0%])	3/8 (37.5%, [−5.8% ~ 80.8%])	0.074
Serological test, IgG (*I*) ≥ 1:800, n/N (% [95% CI])	21/23 (91.3%, [78.8% ~ 103.8%])	9/12 (75.0%, [46.3% ~ 103.7%])	0.313
PCR/NGS (*C. burnetii +*) in tissue, n/N (% [95% CI])	16/19 (84.2%, [66.2% ~ 102.3%])	15/16 (93.8%, [80.4% ~ 107.1%])	0.608
Antibiotic regimen			
Dox + Hyd, n/N (% [95% CI])	18/26 (69.2%, [50.2% ~ 88.2%])	10/17 (58.8%, [32.7% ~ 84.9%])	0.528
Dox + Cip, n/N (% [95% CI])	3/26 (11.5%, [−1.6% ~ 24.7%])	2/17 (11.8%, [−5.3% ~ 28.8%])	1.000
Follow-up time, mean±SD, months	18.9 ± 21.7	16.3 ± 13.7	0.686
Outcomes			
Favorable outcome, n/N (% [95% CI])	17/23 (73.9%, [54.5% ~ 93.3%])	11/16 (68.8%, [43.2% ~ 94.3%])	1.000
Recovery, n/N (% [95% CI])	3/23 (13.0%, [−1.8% ~ 27.9%])	4/16 (25.0%, [1.2% ~ 48.8%])	0.415
Died, n/N (% [95% CI])	2/23 (8.7%, [−3.8% ~ 21.2%])	0/16 (0.0, [0.0 ~ 0.0])	0.503
Ineffective treatment /Relapse, n/N (% [95% CI])	1/23 (4.3%, [−4.7% ~ 13.4%])	1/16 (6.3%, [−7.1% ~ 19.6%])	1.000

Data are presented as n/N, where n is the number of cases with the characteristic and N is the total number of cases with available data for that variable.

When comparing the clinical characteristics of the surgical group and the non-surgical group, we found that only the use of doxycycline combined with ciprofloxacin showed a statistically significant difference between the two groups (*p* < 0.05), and this regimen of doxycycline plus ciprofloxacin was more commonly used in the surgical group. However, all three surgical cases reported in this study received doxycycline combined with ciprofloxacin, which may have a significant impact on the statistical results for the treatment strategy. At the same time, there were no significant differences in the remaining clinical characteristics and outcomes (Table 5).

**Table 5:** Comparing the clinical characteristics, treatment strategies and outcomes of patients with Q fever vertebral osteomyelitis in the surgical group and the non-surgical group.

Item	Surgical group (*n* = 24)	Non-surgical group (*n* = 26)	*P-*value
Age, mean±SD, years	66.3 ± 9.9	60.8 ± 19.5	0.224
Male, n/N (% [95% CI])	21/24 (87.5%, [73.2% ~ 101.8%])	22/26 (84.6%, [69.8% ~ 99.5%])	1.000
Elevated CRP (>5 mg/L), n/N (% [95% CI])	12/17 (70.6%, [46.4% ~ 94.7])	14/18 (77.8%, [56.5% ~ 99.1%])	0.711
Elevated ESR (>15 mm/h), n/N (% [95% CI])	8/13 (61.5%, [30.9% ~ 92.1%])	5/7 (71.4%, [26.3% ~ 116.6%])	1.000
Serological test, IgG (*I*) ≥ 1:800, n/N (% [95% CI])	15/17 (88.2%, [71.2% ~ 105.3%])	19/22 (86.4%, [70.8% ~ 101.9%])	1.000
PCR/NGS (*C. burnetii +*) in tissue, n/N (% [95% CI])	17/18 (94.4%, [82.7% ~ 106.2%])	16/19 (84.2%, [66.2% ~ 102.3%])	0.604
Antibiotic regimen			
Dox + Hyd, n/N (% [95% CI])	14/24 (58.3%, [37.1% ~ 79.6%])	17/25 (68.0%, [48.3% ~ 87.7%])	0.561
Dox + Cip, n/N (% [95% CI])	5/24 (20.8%, [3.3% ~ 38.4%])	0/25 (0.0, [0.0 ~ 0.0])	0.022
Follow-up time, mean±SD, months	17.7 ± 21.5	23.1 ± 23.8	0.461
Outcomes			
Favorable outcome, n/N (% [95% CI])	16/21 (76.2%, [56.3% ~ 96.1%])	14/24 (58.3%, [37.1% ~ 79.6%])	0.342
Recovery, n/N (% [95% CI])	4/21 (19.0%, [0.7% ~ 37.4%])	6/24 (25.0%, [6.3% ~ 43.7%])	0.729
Died, n/N (% [95% CI])	1/21 (4.8%, [−5.2% ~ 14.7%])	1/24 (4.2%, [−4.5% ~ 12.8%])	1.000
Ineffective treatment /Relapse, n/N (% [95% CI])	0/21 (0.0, [0.0 ~ 0.0])	3/24 (12.5%, [−1.8% ~ 26.8%])	0.236

Data are presented as n/N, where n is the number of cases with the characteristic and N is the total number of cases with available data for that variable.

## 4 Discussion

Q fever is a major global zoonotic disease that poses a threat to both public and veterinary health. *C. burnetii* has been detected in various hosts, including humans, domestic and wild animals, pets, birds, and arthropods (46). Given the threats posed by *C. burnetii*, it is crucial to have a comprehensive understanding of its transmission routes and potential sources of infection. In this study, 42% of the patients with Q fever vertebral osteomyelitis had a clear history of contact with cattle, sheep, or wild animals, with cattle and sheep being the most common, and 38% of the patients lived in the countryside. This is broadly consistent with the exposure risk history reported in the literature (47). Not all patients had direct contact with animals, but many lived near dairy farms (48, 49). In a majority of the Q fever outbreak incidents, livestock were the main source of *C. burnetii* infection, primarily transmitted through inhalation of contaminated particles (46). Therefore, even if most cases did not report contact with cattle, goats, or sheep, nor had any experience living around the farm, clinicians should still consider the possibility of Q fever vertebral osteomyelitis.

Q fever can occur in people of all ages, and the age distribution varies slightly across different regions. However, in general, it is still primarily prevalent among the middle-aged and elderly population (47, 50). The average age of patients with Q fever vertebral osteomyelitis in this group is 63.4 ± 15.7 years, which is generally consistent with the mean age of 67 years reported in other studies (35, 42). Q fever vertebral osteomyelitis is more common in male patients, which may be related to occupational differences between genders. The common clinical symptoms of Q fever vertebral osteomyelitis include low back pain and fever. However, clinical manifestations still lack specificity and are difficult to distinguish from spinal tuberculosis and brucellar spondylodiscitis. Moreover, Q fever vertebral osteomyelitis usually affects the lumbar spine, with the L4–L5 level being the most common. However, the most frequently involved vertebra is the L3 segment. Regarding the specific locations where Q fever vertebral osteomyelitis is prone to occur, there are no relevant reports in previous literature.

The clinical manifestations, imaging features, and common affected areas of Q fever vertebral osteomyelitis are highly similar to those of spinal tuberculosis and brucellar spondylodiscitis. These three conditions are often difficult to distinguish from each other. In the past, serological testing has been the main method for diagnosing *C. burnetii* infection (51). However, in this study, the serological positive rate of *C. burnetii* in patients with Q fever vertebral osteomyelitis was not 100%. The indirect immunofluorescence assay for *C. burnetii* phase *I* IgG is the major criterion for Q fever vertebral osteomyelitis; however, it is not the definitive criterion (5, 11). During the effective treatment follow-up of patients with Q fever vertebral osteomyelitis, the serological titer is expected to gradually decrease. However, during the treatment and follow-up of chronic Q fever, serological testing for *C. burnetii* cannot reliably predict disease-related events or treatment failure (52).

The definite criteria for diagnosing *C. burnetii* osteoarticular infection include positive culture or PCR results from affected bone or biopsy tissue (5, 11). However, in this study, the positive rate of spinal lesion tissue culture was extremely low, at only 5.4%. Although culturing and isolating *C. burnetii* can provide definite evidence of infection, this process requires a biosafety level 3 laboratory. Due to the complexity of the technique, high infection risk, and low diagnostic rate, it leads to numerous logistical challenges in clinical practice, thereby limiting its application. Therefore, the main diagnostic criterion currently used in clinical practice continues to rely on the positive PCR results for *C. burnetii* in tissue samples. In this study, the positive PCR detection rate of *C. burnetii* in tissue samples was as high as 86.7%, while the positive rate of NGS was 100%. PCR is the preferred method for confirming *C. burnetii* in clinical samples (53, 54). Metagenomic next-generation sequencing (mNGS) for clinical pathogen detection is becoming more widely available for a variety of sample types and patient indications. The majority of studies have demonstrated that the sensitivity and specificity of mNGS are comparable to those of specific PCR assays, and it can detect more potential pathogens than conventional methods (55). Especially for rare pathogenic bacteria (such as anaerobic bacteria) that cause spinal infections (56), when bacterial culture is difficult to provide a definitive diagnosis, PCR or NGS has significant advantages (55). Furthermore, tNGS is a new method that combines the advantages of PCR and NGS. Through a carefully selected primer pool, tNGS can selectively amplify the target genomic region, enabling the easier detection of target pathogens with high sensitivity while reducing background human DNA and simplifying data complexity. Thus, tNGS is a promising and important tool that can be used for the rapid detection of *C. burnetii* infection (53, 57).

Doxycycline combined with hydroxychloroquine is the gold standard treatment regimen for persistent focal infections caused by *C. burnetii* (5, 11, 58, 59). The number of cases of Q fever osteoarticular infections is limited, and the treatment plan is formulated based on expert recommendations; it has been reported that using doxycycline and hydroxychloroquine for 18 months is a good treatment option (29). The current recommendation is that chronic Q fever should be treated for at least 18–24 months (independent of the focus of infection) (9). Apart from the combination of doxycycline and hydroxychloroquine, doxycycline combined with ciprofloxacin is also an effective alternative option (59). The most common regimen in this study was doxycycline combined with hydroxychloroquine, followed by doxycycline combined with ciprofloxacin. Among them, the regimen of doxycycline plus ciprofloxacin was used more frequently in the surgical group. Of course, the three surgical cases in our study may have had a relatively significant impact on this result. Both regimens can achieve satisfactory therapeutic effects. As van Roeden reported, the efficacy of treating chronic Q fever with tetracyclines combined with quinolones is not inferior to that of tetracyclines combined with hydroxychloroquine (60). Currently, the regimen of doxycycline combined with hydroxychloroquine remains the most extensively studied clinical approach for treating chronic Q fever. However, retrospective studies have not corroborated a clear benefit of this combination, particularly in comparison with other regimens (61). At present, the medical community has not reached a consensus on this issue, so conducting high-quality controlled trials is extremely necessary. However, conducting prospective studies on rare diseases is extremely challenging. This challenge extends to the international level because of the rarity, heterogeneity, and the prolonged course of these diseases.

The indications for surgical treatment of spinal lesions include neurological deficits, spine instability and/or deformity, the presence of epidural abscess, and failure of conservative treatment (62). A review of the cases revealed that nearly half of the patients underwent debridement surgery, but there was no significant difference in prognosis and outcomes between the surgical group and the non-surgical group. For Q fever vertebral osteomyelitis, antibiotic treatment remains the cornerstone, while surgery serves as a necessary adjunctive measure. For patients with definite needs, surgical treatment is also a very important supplementary treatment option.

Furthermore, 60.5% of the patients with Q fever vertebral osteomyelitis also had vascular comorbidities, among which AAA was the most common. This is in complete agreement with the data reported by Alder and Liu (7, 42), which indicated that the proportion of patients with osteoarticular involvement due to *C. burnetii* infection and accompanied by aortic aneurysm was 60–61.5%. The case review in this study revealed that the majority of patients had a history of aneurysms or related surgical procedures before presenting with clinical manifestations related to *C. burnetii* infection. However, there is also a view that aneurysms are the most common complications of chronic Q fever (63). Currently, there are no clear research reports confirming that patients with aneurysms are more prone to infection with *C. burnetii* or that patients infected with *C. burnetii* are more likely to develop aneurysms. Therefore, for patients with spinal infections accompanied by aneurysms, if the specific type of infection cannot be determined, clinicians should highly suspect Q fever vertebral osteomyelitis caused by the rare pathogen *C. burnetii* as a possibility.

In the subgroup analysis of Q fever vertebral osteomyelitis with vascular lesions versus simple Q fever vertebral osteomyelitis, the results showed no significant differences in treatment strategies and outcomes between the two groups. However, all the deceased patients had vascular lesions. The reported mortality rate of Q fever was 2.0% (47), but the mortality rate of patients with infected aortic aneurysm due to *C. burnetii* can be as high as 25% (64). The findings of Botelho-Nevers indicate that surgery is crucial in the management of vascular infections caused by *C. burnetii*, and it has a particularly significant association with recovery (64). Therefore, for patients with Q fever vertebral osteomyelitis accompanied by vascular infections, in addition to antibiotic treatment, surgical treatment of the infected aneurysms or vascular grafts is also an essential management measure.

## 5 Limitations

This study is a retrospective study based on existing evidence, and it is subject to publication bias and reporting bias. The included cases are heterogeneous, and many published reports have omitted some clinical variables. Due to the rarity of the disease, robust statistical comparisons were not possible.

## 6 Conclusion

Q fever vertebral osteomyelitis should be included in the differential diagnosis, particularly in older men and patients with concurrent vascular abnormalities. PCR or NGS performed on local specimens provides critical advantages for rapid pathogen identification. Doxycycline combined with hydroxychloroquine remains the most used treatment regimen at present.

## Data Availability

The original contributions presented in the study are included in the article/supplementary material, further inquiries can be directed to the corresponding author/s.
